# Economic and Disease Burden of Dengue in Southeast Asia

**DOI:** 10.1371/journal.pntd.0002055

**Published:** 2013-02-21

**Authors:** Donald S. Shepard, Eduardo A. Undurraga, Yara A. Halasa

**Affiliations:** Schneider Institutes for Health Policy, Heller School for Social Policy and Management, Brandeis University, Waltham, Massachusetts, United States of America; Duke University-National University of Singapore, Singapore

## Abstract

**Background:**

Dengue poses a substantial economic and disease burden in Southeast Asia (SEA). Quantifying this burden is critical to set policy priorities and disease-control strategies.

**Methods and Findings:**

We estimated the economic and disease burden of dengue in 12 countries in SEA: Bhutan, Brunei, Cambodia, East-Timor, Indonesia, Laos, Malaysia, Myanmar, Philippines, Singapore, Thailand, and Viet Nam. We obtained reported cases from multiple sources—surveillance data, World Health Organization (WHO), and published studies—and adjusted for underreporting using expansion factors from previous literature. We obtained unit costs per episode through a systematic literature review, and completed missing data using linear regressions. We excluded costs such as prevention and vector control, and long-term sequelae of dengue. Over the decade of 2001–2010, we obtained an annual average of 2.9 million (m) dengue episodes and 5,906 deaths. The annual economic burden (with 95% certainty levels) was US$950m (US$610m–US$1,384m) or about US$1.65 (US$1.06–US$2.41) per capita. The annual number of disability-adjusted life years (DALYs), based on the original 1994 definition, was 214,000 (120,000–299,000), which is equivalent to 372 (210–520) DALYs per million inhabitants.

**Conclusion:**

Dengue poses a substantial economic and disease burden in SEA with a DALY burden per million inhabitants in the region. This burden is higher than that of 17 other conditions, including Japanese encephalitis, upper respiratory infections, and hepatitis B.

## Author Summary

Dengue fever, or break bone fever, is the most common infectious disease transmitted by a mosquito, and is a major economic and disease burden in endemic countries. Between 100–200 million (m) infections occur each year in more than 100 countries, resulting in about 20,000 deaths. Quantifying the burden of dengue is critical for policy makers to set policy priorities and make informed decisions about disease control. We estimated the economic and disease burden of dengue in 12 countries in Southeast Asia, using a consistent methodology that allows comparison among countries. We estimated an annual average of 2.9 m dengue episodes and 5,906 deaths. This amounts to an annual cost per capita of US$1.65 (0.03% GDP per capita in 2010), and a disease burden of 372 disability-adjusted life years (DALYs) per million inhabitants, a rate higher than that of 17 other conditions, including Japanese encephalitis, upper respiratory infections, and hepatitis B.

## Introduction

Dengue fever is among the most important infectious diseases in tropical and subtropical regions of the world, and represents a significant economic and disease burden in endemic countries [1]–[4]. There are about 100–200 million infections per year in more than 100 countries [5]. Estimating the economic and disease burden of dengue is critical to inform policy makers, set health policy priorities, and implement disease-control technologies.

Here we estimate the economic and disease burden of dengue in 12 countries of Southeast Asia (SEA). We included all countries in the Association of Southeast Asian Nations [6], plus Bhutan and East-Timor due to their geographic proximity, to be consistent with our study on the incidence of dengue in the region [7]. Our study area comprises the following 12 countries: Bhutan, Brunei, Cambodia, East-Timor, Indonesia, Laos, Malaysia, Myanmar, Philippines, Singapore, Thailand, and Viet Nam. Studying dengue burden in SEA is important for several reasons. Dengue is among the greatest disease burdens in SEA, and has been hyperendemic for decades [8]–[11]. SEA is the region with the highest dengue incidence, with cycles of epidemics occurring every three to five years [1], [8]. The WHO regions of SEA and the Western Pacific represent about 75% of the current global burden of dengue [12], [13].

Recent studies have estimated economic burden of dengue in specific countries of SEA (costs in 2010 US dollars [14]). For example, using the average reported cases between 2001–2005, Suaya et al. [2] estimated that the annual costs for dengue illness (standard errors in parenthesis) in Cambodia, Malaysia, and Thailand were at least US$3.1 (±0.2), US$42.4 (±4.3), and US$53.1 (±11.4) million (m), respectively. Beaute and Vong estimated an annual cost (2006–2008) of US$8.0m for Cambodia [15]. Adjusting the officially reported cases in 2009 with expansion factors (EFs) derived from a Delphi process, Shepard et al. [16] estimated that the annual cost of dengue in Malaysia, as updated [17], was about US$103.4m per year (range: US$78.8m–US$314.2m). Lim et al. [18] estimated a yearly cost of dengue–including dengue illness, vector control, and research and development activities–of US$133m (range: US$88m–US$215m) in Malaysia (2002–2007) and US$135m (range: US$56m–US$264m) in Thailand (2000–2005), respectively, in which dengue illness represented about 41.3% of the total costs (US$54.9m) in Malaysia and 49% (US$66.2m) in Thailand. Based on data from a provincial hospital, Kongsin et al. [19] estimated that the total economic burden of dengue in Thailand was US$175.4m (standard deviation: US$36.6m), of which US$126.3m corresponded to dengue illness and US$49.1m to dengue control. In Singapore, Carrasco et al. [20] estimated that yearly dengue illness costs US$41.5m and vector control costs US$50.0m. Last, Luong et al. [21] obtained an average annual cost (2004–2007) of US$30.3m for Viet Nam. The dengue burden of disease (number of disability adjusted life years or DALYs, based on the original 1994 definition [22] and extrapolated to 2010 based on population) has also been estimated for Cambodia (8,200 [15]), Myanmar (3,900 [23]), Singapore (700 [20]), and Thailand (28,900 [24]; 32,500 [25]).

The few published estimates of economic and disease burden of dengue in SEA are based on a single or a small number of countries, and the comparison of estimates is limited by methodological differences between studies. Previous multi-country studies of dengue burden include the economic impact of dengue in the Americas [3], and an eight-country study including five countries in the Americas and three in SEA [2]. This paper aims to reduce this gap by estimating the economic and disease burden of dengue illness in SEA using a consistent methodology.

## Methods

The economic burden of dengue is calculated as the total number of dengue cases times the total costs per dengue episode. To calculate the disease burden, an estimate of the total DALY burden per cases is also required.

### Total number of dengue cases

Because dengue is an infectious disease, there is considerable annual variability in the number of dengue cases. We used the average officially reported cases in 2001–2010 to obtain a more stable estimate for each country. We obtained the number of reported dengue cases from various sources, including data from the country's Ministry of Health or statistics agency, WHO, or published studies [12], [16], [26]–[35]. Dengue is a reportable illness in SEA and thus the number of cases reported is correlated to the total cases. However, there is substantial underreporting of symptomatic dengue fever in SEA, and official statistics commonly underestimate case rates [7], [36].

Estimating the total number of dengue cases is challenging due to the limits of passive surveillance systems, which are useful to detect dengue outbreaks and to understand long-term trends of symptomatic infection, but underestimate the true incidence. The rate of reporting of surveillance systems depends on several variables, including the severity of dengue, identification method (e.g., clinical diagnosis, laboratory test), treatment facilities, year of data collection, the area where dengue is measured, among others [16], [27]. Recent studies have improved the estimate of the total number of cases by using EFs [3], [7], [16], [20], the ratio of the best estimate of the total number of symptomatic dengue, divided by the number of reported cases.

We adjusted the officially reported cases using Undurraga et al.'s estimates of EFs for ambulatory, hospitalized, and total dengue episodes to estimate the incidence of dengue by country [7]. Undurraga et al. estimated the annual average of dengue episodes based on the officially reported cases from 2001 through 2010, and derived country-specific EFs through a systematic analysis of published studies that reported original, empirically derived EFs or the necessary data to obtain them.

### Costs per dengue episode

To estimate the economic burden of symptomatic dengue infection one requires information on the unit costs of providing inpatient and outpatient medical care, in both private and public facilities. We conducted a systematic literature review for articles on the economic costs of dengue in Southeast Asia published between 1995 and 2012 using Web of Science and MEDLINE (72 articles), and PubMed (97 articles) using the keywords dengue, health, and economics. We reviewed the abstracts of these articles and identified 11 articles that explicitly reported data on the economic costs per dengue fever episode, or included the necessary information to estimate them [2], [15], [23], [24], [37]–[43]. To these articles, we added nine recently published articles [16], [19], [20], [44], or found in previous searches [21], [25], [45]–[47]. Although this study is an original research study and not a systematic review, we adapted relevant parts of the PRISMA check list and flowchart to our literature review (Figure S1, Table S1) [48].

We then filtered these 20 articles based on the following criteria: (1) use of original, empirical data; (2) use of a scientifically consistent approach; (3) use of externally valid and representative data; and (4) use of recent data in order to reflect current medical practice and technology. We selected studies that scored well, albeit not perfectly, on these criteria, providing what we think are the best data available. For each of these countries we derived the best cost estimate for direct medical and non-medical costs and indirect costs, for both inpatient and outpatient treatment. For countries in which no cost data were available, we relied instead on expert opinion (Malaysia) or in the extrapolation of data based on regression analysis (Bhutan, Brunei, East Timor, Indonesia, Laos, Myanmar, and Philippines), using unit costs as the dependent variable and gross domestic product (GDP) per capita as the independent variable.

We found six studies that included dengue costs for Cambodia [2], [15], [37], [39], [40], [44]. Our best estimates for direct costs are based on the average between the costs estimates of two studies by Suaya et al. [39], [44]; to estimate indirect costs we used an average between these two studies plus the estimates by Huy et al. [37]. In the first study, Suaya et al. estimated costs based on patient interviews and record reviews of hospitalized patients from Daun Keo Referral Hospital [44]. In the second study considered, the authors' estimates were based on expert opinion and interviews with families, and contrasted with survey data from hospitalized patients and financial data from the National Pediatric Hospital [39]. Two additional studies estimated out-of-pocket expenditures, which may not necessarily reflect the real costs of a dengue episode [37], [40]. We used Huy et al.'s estimates to obtain indirect costs per dengue episode [37]. As Beaute and Vong's estimates were based on secondary analysis of data, they were excluded [15].

For Viet Nam, our best cost estimates were based on the results from an unpublished multicenter cost study in southern Viet Nam by Luong et al. [21], which included data on medical expenditures from four hospitals, transportation costs, and household impact. Patients were recruited based on severity, age, and type of setting, and adjusted the costs accordingly. Another study based on Viet Nam also provided detailed data on dengue; however, it was restricted only to dengue hemorrhagic fever (DHF) cases in children <15 years from a single hospital [43].

The costs for Malaysia were estimated based on a previous study of the unit costs of inpatient and outpatient hospital services at the University of Malaya Medical Center (UMMC) in 2005, reported by Suaya et al. [2]. Shepard and others [16], [17] updated these unit costs estimates by including salaries for academic clinicians not captured in 2005. These authors then adjusted the unit costs for inpatient and outpatient dengue cases using a weighted average by type of setting – primary, secondary, and tertiary hospitals – based on WHO-Choice estimates [47]. Our best cost estimates for Malaysia were based on Shepard et al.'s [16], [17] update of the UMMC study.

We found five studies including cost estimates for Thailand [2], [19], [25], [41], [42]. Our best cost estimates were based on a study by Kongsin et al. [19], which used the same cost data as Suaya et al. [2]. These data included direct and indirect costs obtained from interviews and medical records from a provincial hospital. Their cost estimate per outpatient visit was calculated as 25% of the costs of an inpatient bed-day (based on Shepard et al. [49]). The study by Okanurak et al. [42] was not included because the data were too old (1994). Because health costs have been increasing in the past decades, among other reasons due to changes in technology and treatments, adjusting Okanurak et al.'s data for inflation would most likely have underestimated the cost per dengue episode. Other studies reviewed were by Anderson et al. [25], whose estimates were based on expert opinion, previous analysis in the region, and discussion with a subset of families, and by Lee et al. who used secondary data [41].

Last, the costs for Singapore were based on Carrasco et al.'s estimates of the direct and indirect costs of dengue from inpatient and outpatient cases [20]. The direct costs of hospitalization were obtained from the distribution of hospital bills per dengue patient provided by public Singaporean hospitals in 2010 for unsubsidized wards, divided by the median length of stay. The costs of ambulatory cases were obtained by multiplying the average number of visits per case by the unit costs of each visit. The study also included non-medical indirect costs.

For those countries for which we could not obtain empirical data, we extrapolated direct and indirect costs per non-fatal dengue episode using bivariate regressions for each type of cost. We used ln(cost)-direct and indirect cost-as the dependent variables, and ln(GDP per capita) and a dummy variable for hospitalized and ambulatory patients as independent variables. The regressions included robust standard errors and clustering by country.

Last, we estimated the indirect costs per fatal episode using the human capital approach -based on productivity loss- and estimated the total years of premature life lost based on the discounted, weighted life expectancy using WHO life tables for each country [50], [51]. As data on the age distribution of fatal cases was not available, we assumed it followed the distribution of all dengue cases (except for Malaysia, for which we used the age distribution of fatal episodes in 2009 [30]). We obtained country-specific age distributions of dengue cases from various sources, including surveillance data and published studies [15], [21], [30], [32], [34], [35], [52]–[56]. We interpolated values when data did not include the specific age ranges, and used the regional average when country-specific age distributions were not available. For the age of fatal cases, we used the midpoint of each closed interval (0–4, 5–14, 15–29, 30–44, 45–59), and age 65 for the highest category (60+). As in previous studies [2], [3], we valued years lost based on the country's per capita GDP [14] and discounted at a real rate of 3% per year.

### Disease burden of dengue (DALYs)

We estimated the disease burden of dengue using WHO methodology [57], and expressed disease burden in standard DALYs. For fatal cases, we used the same age patterns as for indirect costs. To account for disability during non-fatal dengue cases, we considered an average duration of 14 days for hospitalized patients (range 10–18), and 4.5 days for ambulatory patients (range 2–7), and a disability weight of 0.81 for both hospitalized and ambulatory cases, as in previous studies [3], [58], [59]. To compare the burden of dengue with that of other diseases in the region, we combined the two WHO regions (Southeast Asia and Western Pacific) containing the 12 countries considered here by adding the populations and respective DALYs by cause. We then calculated the overall DALY burden per million population for each of the 39 individual causes of death or disability (excluding subtotals) reported by WHO in its Annex Table A2 [57].

### Sensitivity analysis

We ran 1,000 Monte Carlo simulations for a probabilistic analysis of the total costs of dengue illness, simultaneously varying four parameters: (1) EFs, (2) the share of the total dengue cases treated in hospitals; (3) the unit costs per dengue case, and (4) DALYs per dengue case. We varied EFs based on Undurraga et al.'s sensitivity analysis [7], using triangular distributions based on country-specific estimates, as shown in Table 1. Country-specific triangular distributions were used to represent the variation in unit costs. We considered our best estimates as the mode (Table 2), and estimated the variation of unit costs considering the same variability of costs estimated by WHO-Choice (World Health Organization – CHOosing Interventions which are Cost-Effective) [47]. Last, we accounted for DALYs variation using a uniform distribution.

**Table 1:** Reported and estimated dengue episodes in Southeast Asia, annual average (2001–2010).

Country,	Reported cases		Expansion factors a			Estimated dengue episodes b			Estimated
(ISO code)	All	Fatal	Hospitalized	Ambulatory	Overall	Hospitalized	Ambulatory	All	Deaths^b^
Bhutan^c^	67	2	2.5	n.r	12.9	168	699	866	5
(BTN)			(1.0–3.4)		(9.5–20.3)	(80–200)	(504–1,049)	(657–1,175)	(3–7)
Brunei	72	0	2.5	6.2	4.9	65	286	351	1
(BRN)			(1.0–3.4)		(4.4–5.5)	(31–76)	(237–307)	(299–356)	(0–1)
Cambodia	14,407	147	1.8	n.r.	12.9	26,399	159,451	185,850	269
(KHM)			(0.6–3.0)		(3.9–29.3)	(11,402–72,047)	(46,430–318,932)	(84,896–353,752)	(122–774)
East Timor^c^	323	5	2.5	n.r.	19.0	808	5,330	6,137	13
(TLS)			(1.0–3.4)		(11.5–54.6)	(517–1,331)	(3,299–13,251)	(4,428–14,195)	(6–15)
Indonesia	104,457	1,041	3.3	n.r.	7.6	344,708	448,121	792,829	3,436
(IDN)			(1.0–3.4)		(7.1–9.9)	(142,183–346,978)	(443,306–769,784)	(761,886–988,820)	(1,417–3,459)
Laos	8,536	17	2.5	56.8	11.3	17,790	78,758	96,548	41
(LAO)			(1.0–3.4)		(8.8–15.9)	(8,906–21,266)	(58,495–105,366)	(76,172–119,812)	(21–50)
Malaysia	37,886	95	1.7	65.6	3.8	62,256	81,635	143,891	162
(MYS)			(1.0–3.4)		(2.5–6.2)	(42,561–108,311)	(15,084–146,645)	(100,499–206,432)	(114–291)
Myanmar^c^	15,313	149	2.5	n.r.	16.2	38,283	209,660	247,943	372
(MMR)			(1.0–3.4)		(10.7–33.6)	(17,971–45,538)	(138,603–408,363)	(173,385–437,328)	(172–436)
Philippines	45,409	487	2.5	11.7	7.0	58,207	257,685	315,892	1,218
(PHL)			(1.0–3.4)		(6.2–7.9)	(28,098–68,905)	(215,178–283,749)	(269,854–325,239)	(595–1,459)
Singapore	6,362	10	2.5	5.0	4.1	8,986	17,352	26,339	26
(SGP)			(1.0–3.4)		(1.0–4.9)	(6,091–14,734)	(2,172–19,285)	(9,529–28,304)	(13–32)
Thailand	76,978	98	2.9	29.8	8.5	176,357	481,455	657,811	285
(THA)			(0.8–8.7)		(8.0–12.5)	(125,716–530,046)	(149,232–661,085)	(602,752–861,356)	(207–874)
Viet Nam	76,364	241	1.2	n.r.	5.8	81,611	361,300	442,911	80
(VNM)			(1.0–3.4)		(5.4–6.7)	(80,001–218,672)	(202,851–369,179)	(397,859–470,849)	(82–224)
Total	386,154	2,126	2.4	n.r.	7.6	815,636	2,101,732	2,917,368	5,906
			(2.1–2.9)		(7.0–8.8)	(457,493–1,408,647)	(1,245,867–3,068,345)	(2,437,421–3,760,035)	(2,719–7,489)

Notes: ISO Alpha-3 codes were obtained from United Nations [60]; estimated lower and upper ranges are shown parenthesis; n.r. denotes not reported.

a Estimates for expansion factors (EFs) based on Undurraga et al. [7].

b Point estimates were obtained by multiplying the average reported episodes of dengue in 2001–2010 by the corresponding EF, as reported by Undurraga et al. [7]. The range in parentheses for the total hospitalized and ambulatory dengue episodes and deaths corresponds to the 95% certainty level using 1,000 Monte Carlo simulations. We varied EFs using triangular distributions based on [7], and assumed that the EF for the total deaths was the same as the EF for hospitalized dengue episodes. While more severe episodes of dengue are more likely to be reported, there is some evidence of underreporting of severe dengue resulting in death [61], and recent studies suggest that dengue is associated with several health complications [62]–[67]. We expect that some of these resulting deaths would not be reported as dengue.

c Officially reported cases of dengue were available until September 2010, the number of cases for the remaining months were extrapolated based on comprehensive surveillance data from Thailand and Indonesia [31], [32], assuming that the time distribution of dengue episodes was similar.

Sources: [7], [12], [16], [17], [26], [27], [29]–[35], [60].

**Table 2:** Unit cost per dengue episode from original studies, by country (2010 US$).

			Inpatient episodes				Outpatient episodes			
Country	Source	Setting	Direct (Med.)	Direct (Non-med.)	Indirect	Total Inpatient	Direct (Med.)	Direct (Non-med.)	Indirect	Total Outpatient
*(1) Low income countries*										
Cambodia	Suaya et al. [39]	Secondary level	64.29	36.29	21.75	122.33	7.72	11.10	-	18.82
	Suaya et al. [44] a	Secondary level	31.08	36.63	61.05	128.76	n.a.	n.a.	n.a.	n.a.
	Huy et al. [37] b	Various settings	21.21	9.03	13.00	43.25	7.57	3.22	4.64	15.42
	*Best estimate*		*47.69*	*36.46*	*31.93*	*116.08*	*7.72*	*11.10*	*4.64*	*23.46*
*(2) Lower-middle income countries*										
Viet Nam	Harving et al. [43] c	Tertiary	36.33	17.59	14.18	68.10	n.a.	n.a.	n.a.	-
	Luong et al. [21] d	All settings	46.66	17.00	12.75	76.41	9.80	11.81	9.85	31.46
	*Best estimate*		*46.66*	*17.00*	*12.75*	*76.41*	*9.80*	*11.81*	*9.85*	*31.46*
*(3) Upper-middle income countries*										
Malaysia	Shepard et al.[16], [17]	All settings	598.81	61.10	203.31	863.21	221.78	22.40	178.02	422.20
	Suaya et al. [2]	Tertiary level	834.31	85.12	131.65	1,051.07	214.66	21.68	115.79	352.12
	*Best estimate*		*598.81*	*61.10*	*203.31*	*863.21*	*221.78*	*22.40*	*178.02*	*422.20*
Thailand	Okanurak et al. [42]	All settings	118.55^e^	24.67	56.71^e^	199.93	n.a.	n.a.	n.a.	-
	Kongsin et al. [19] f	Tertiary	518.33	66.59	49.95	634.87	129.58	16.65	12.49	158.72
	*Best estimate*		*518.33*	*66.59*	*49.95*	*634.87*	*129.58*	*16.65*	*12.49*	*158.72*
*(4) High-income countries*										
Singapore	Carrasco et al. [20]	Tertiary level	2,025.70	34.78	947.99^g^	3,008.47	368.89	26.03	873.36^g^	1,268.28
	*Best estimate*		*2,025.70*	*34.78*	*947.99*	*3,008.47*	*368.89*	*26.03*	*873.36*	*1,268.28*

Notes: n.a. denotes not available; med. denotes medical; UMMC: University of Malaya Medical Center.

a The cost data from Suaya et al. [44] is the same as the data from Suaya et al. [2]; we included only the most recently published.

b The costs estimated by Huy et al. [37] were out-of-pocket expenses, based on a standardized questionnaire. Indirect costs included income lost due to days of work lost when caring for the child, or to pay for this care.

c The costs were based on out-of-pocket expenses by patients' households. Patients were hospitalized children aged 0–15 years with DHF.

d Luong et al. [21] recruited patients based on severity, age, and type of setting. To obtain an estimate of the costs, they adjusted the data considering that (i) 65% of the reported cases of dengue in Southern Viet Nam from 2004 to 2007 were children<15 years old, (ii) the distribution of severity of cases corresponds to that reported by Tien et al. [69] (inpatient: 13% dengue fever (DF), 87% dengue hemorrhagic fever (DHF) & dengue shock syndrome (DSS); outpatient: 100% DF), and (iii) that the distribution of cases by setting is proportional to the number of beds in the study area (i.e., for children: 41% referral, 27% provincial, 31% district; for adults: 0% referral, 69% provincial, 31% district).

e The estimated cost is the average between the costs per bed-day in a hospital in Bangkok and in Suphan Buri, and corresponds to DHF patients. We assumed that 45% of patients were adults (>15 yrs) based on data by the National Surveillance System (2004–2010).

f The data by Kongsin et al. [19] are the same as the data used by Suaya et al. [2]. The costs per ambulatory case were estimated as 25% of those per hospitalized case based on Shepard et al. [49].

g Estimate for patients aged 18–64 years based on transport costs, average productivity loss per day, and household services lost per day. For hospitalized patients, the estimate considers the average number of days a person is hospitalized per dengue episode, and for ambulatory patients, the total number of visits per episode.

## Results

The average annual number of reported cases in SEA was 386,000 patients (2001–2010), and 2,126 deaths. Using corresponding EFs, we obtained a yearly average of about 2.9 m cases of dengue illness in SEA (0.8 m hospitalized and 2.1 m ambulatory patients), 5,906 deaths, and a weighted overall EF of 7.6. Table 1 shows the annual average number of reported dengue cases in SEA (2001–2010), the estimated hospitalized, ambulatory, and total number of dengue cases, and the total number of deaths, using country-specific EFs. The lower and upper ranges for each of our estimates are shown in parentheses.

Our literature review yielded 20 studies on unit costs per dengue episode [2], [15], [16], [19], [21], [23]–[25], [37]–[47]. We extracted data from the articles using a template similar to Table 2, with additional columns (e.g., date the article was reviewed, limitations). After applying our filtering criteria, we had sound data for five countries-Cambodia, Viet Nam, Malaysia, Thailand, and Singapore-one for each category of income-level defined by the World Bank (e.g., low-income country) [68], which makes our extrapolated estimates more consistent. Table 2 shows a summary of our best estimates for the unit costs per dengue episode for each country (2010 US dollars). While the summary data may not necessarily be representative of each country, to our knowledge they are the best cost data available.

**Figure 1:**
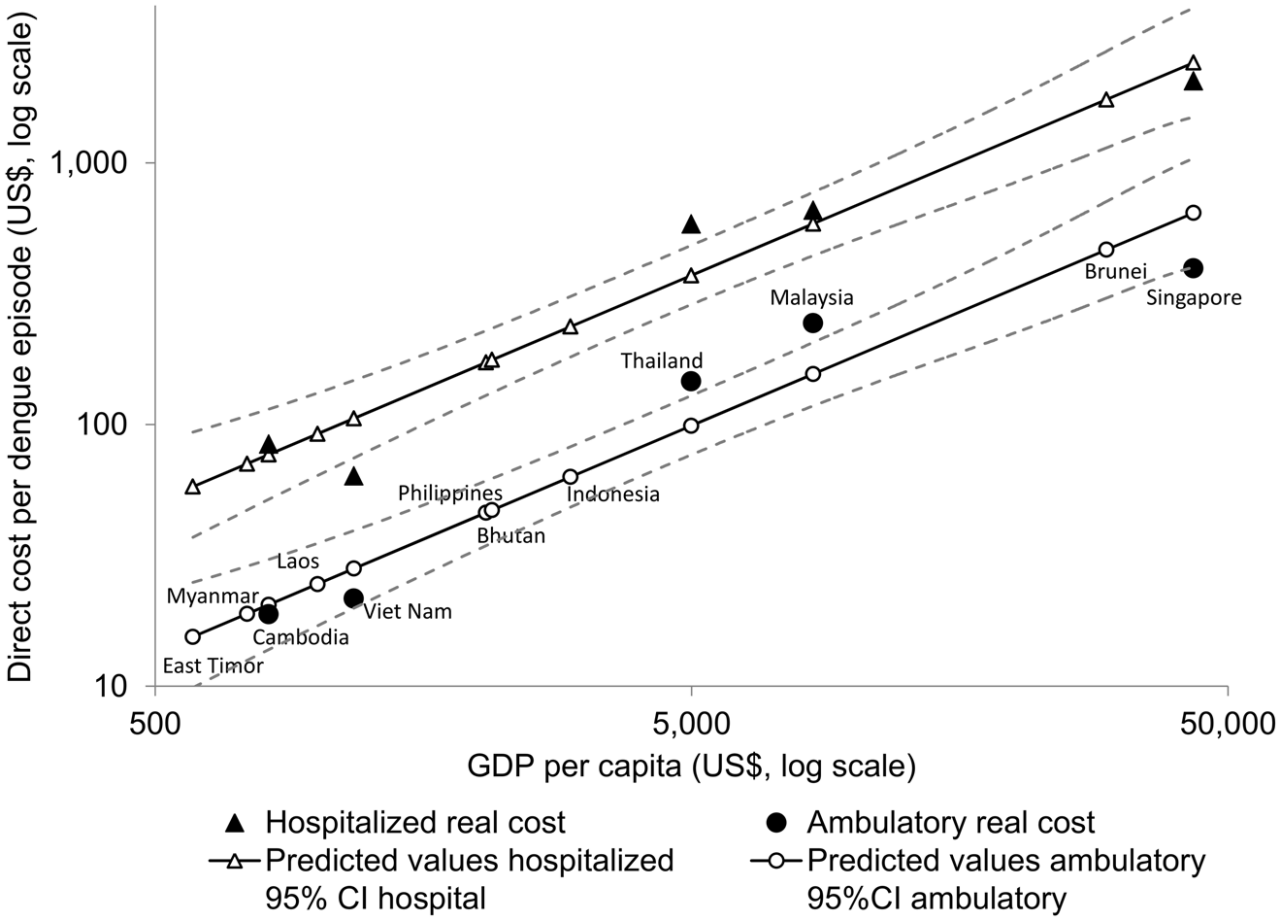
Direct costs per non-fatal dengue episode for hospitalized and ambulatory cases by per capita GDP (2010 US$). Source: Authors' calculations from [2], [16], [17], [19]–[21], [37], [39], [42]–[44], [47].

**Figure 2:**
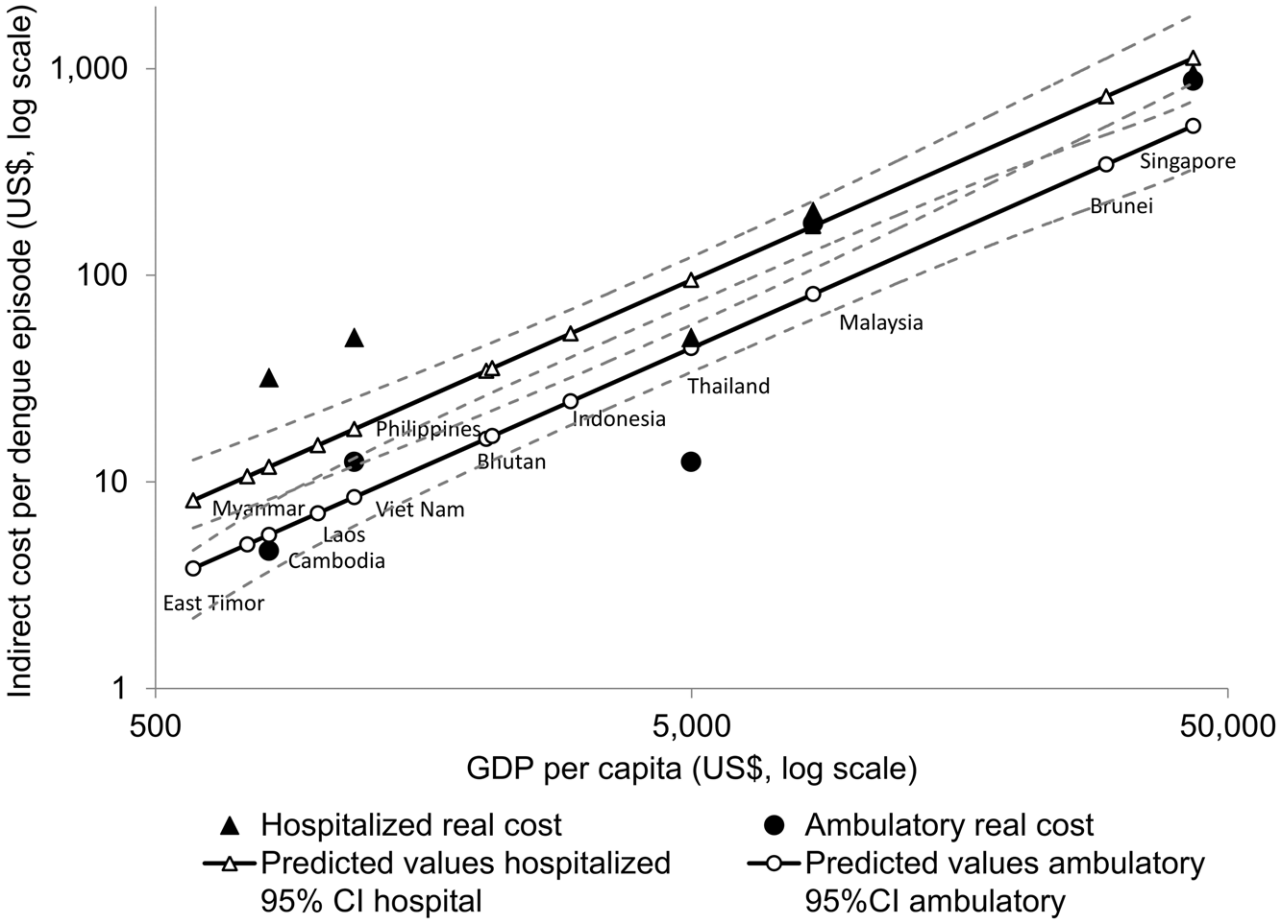
Indirect costs per non-fatal dengue episode for hospitalized and ambulatory cases by per capita GDP (2010 US$). Source: Authors' calculations from [2], [16], [17], [19]–[21], [37], [39], [42]–[44], [47].

Table 3 shows the predicted values of direct and indirect unit costs per dengue case based on the linear regression estimates (R^2^ = 0.94 and 0.87, respectively), for those countries for which we did not have empirical data. Figure 1 and Figure 2 show the relation between GDP per capita and unit direct and indirect costs per episode respectively, and the 95% CI for each set of estimates.

**Table 3:** Predicted values of direct and indirect unit costs per dengue case, based on linear regression estimates (2010 US dollars).

Country	GDP per capita	World Bank classification	Direct Costs		Indirect Costs	
			Hosp.	Amb.	Hosp.	Amb.
Bhutan	2,010	Lower-middle	172.8	46.1	34.5	16.2
Brunei	28,832	High	1,747.4	465.8	733.6	343.9
Cambodia^a^	791^b^	Low	84.1	18.8	31.9	4.6
East Timor	571^b^	Lower-middle	57.9	15.4	8.1	3.8
Indonesia	2,890	Lower-middle	236.8	63.1	52.3	24.5
Laos	976^b^	Lower-middle	92.2	24.6	15.0	7.0
Malaysia^a^	8,184	Upper-middle	659.9	244.2	203.3	178.0
Myanmar	721^b^	Low	70.9	18.9	10.6	5.0
Philippines	2,063	Lower-middle	176.7	47.1	35.5	16.6
Singapore^a^	41,893^b^	High	2,060.5	394.9	948.0	873.4
Thailand^a^	4,850	Upper-middle	584.9	146.2	50.0	12.5
Viet Nam^a^	1,141^b^	Lower-middle	63.7	21.6	12.7	9.9

a Unit costs were obtained from empirical data and not from extrapolation.

b International Monetary Fund (IMF) estimate for 2010.

Notation: GDP denotes gross domestic product; Hosp. denotes Hospitalized; Amb. denotes Ambulatory.

Source: IMF [14]; World Bank [68]; and cost data sources shown in Table 2 [2], [16], [17], [19]–[21], [37], [39], [42]–[44], [47].

### Economic and disease burden of dengue in SEA

Table 4 shows the average total annual economic and disease burden of dengue by country. The table includes the 95% certainty level bounds obtained using 1,000 Monte Carlo simulations in parenthesis under each estimate. Using our best estimates for the total number of cases and the unit cost per dengue episode, we obtained an overall annual economic burden of dengue of US$950 million (m) (US$610m–US$1,384m). The average annual direct costs amounted to US$451m (US$289m–US$716m) and the indirect costs were US$499m (US$290m–US$688m). Indonesia was the country with the highest economic burden of dengue in the region, followed by Thailand, representing about 34% and 31% of the total economic burden of dengue, respectively. The average population for SEA in the years considered was about 574 m people [70]–[72]; hence the cost of dengue illness was about US$1.65 per capita (US$1.06–US$2.41). The costs per capita by country ranged from US$0.28 (US$0.19–US$0.39) in Viet Nam to US$14.99 (US$9.37–US$21.10) in Singapore.

**Table 4:** Annual dengue economic and disease burden in DALYs, by country (average, 2001–2010).

Country	Population (1,000 s)	Aggregate costs (2010 US$, 1,000 s)			Cost per capita (2010 US$)	DALYS
		Direct	Indirect	Total		
Bhutan	726	59	238	295	0.41	148
		(39–84)	(135–319)	(183–389)	(0.25–0.54)	(86–198)
Brunei	378	223	412	636	1.69	14
		(154–296)	(268–520)	(441–802)	(1.17–2.12)	(9–19)
Cambodia	13,670	6,264	10,317	16,540	1.21	15,452
		(2,899–10,663)	(3,890–19,558)	(7,763–29,598)	(0.57–2.17)	(5,910–29,202)
East Timor	1,061	163	199	363	0.34	417
		(90–284)	(119–257)	(231–529)	(0.22–0.50)	(249–563)
Indonesia	232,462	93,470	229,199	323,163	1.39	95,168
		(64,017–130,726)	(127,273–281,114)	(205,440–407,748)	(0.88–1.75)	(52,759–117,836)
Laos	5,931	3,427	1,654	5,093	0.86	2,369
		(2,273–4,643)	(1,154–2,125)	(3,592–6,717)	(0.61–1.13)	(1,457–3,162)
Malaysia	27,051	64,426	63,431	127,973	4.73	8,324
		(47,195–98,585)	(48,377–89,790)	(90,478–181,432)	(3.34–6.71)	(5,517–12,393)
Myanmar	46,916	6,917	7,607	14,476	0.31	13,620
		(4,094–10,841)	(4,675–10,083)	(9,393–20,006)	(0.20–0.43)	(8,006–18,205)
Philippines	88,653	20,656	60,740	80,829	0.91	37,685
		(14,685–27,365)	(35,148–79,301)	(52,126–103,948)	(0.59–1.17)	(22,089–49,617)
Singapore	4,476	25,156	42,076	67,090	14.99	1,089
		(14,363–38,944)	(26,751–56,578)	(41,946–94,430)	(9.37–21.10)	(660–1,509)
Thailand	67,796	215,722	74,303	290,028	4.28	28,475
		(134,028–375,270)	(39,335–139,060)	(181,559–505,186)	(2.68–7.45)	(16,505–49,552)
Viet Nam	85,007	14,814	8,659	23,453	0.28	11,079
		(10,103–21,468)	(6,269–11,890)	(16,463–33,099)	(0.19–0.39)	(7,226–16,452)
Total	574,236	451,297	498,836	949,940	1.65	213,839
		(289,492–715,924)	(290,043–688,415)	(609,614–1,383,882)	(1.06–2.41)	(120,472–298,709)

Note: Cost estimates and their corresponding 95% certainty levels (in parentheses), were obtained using 1,000 Monte Carlo simulations with the simultaneous variation of expansion factors (EFs), the share of hospitalized cases, unit costs for ambulatory and hospitalized cases, and disability-adjusted life years (DALYs).

We obtained an annual average of 214,000 DALYs (range: 120,000–299,000 DALYs) for SEA (Table 4), which is equivalent to 372 DALYs per million inhabitants (range: 210–520). About 45% of the total disease burden in the region is incurred by Indonesia, followed by the Philippines with about 18% of the total. Using the original 1994 definition [22], the rate of DALYs per million population for dengue in SEA ranks higher than that of 17 of the 39 health conditions in SEA and the Western Pacific combined, including poliomyelitis (1 per m), Japanese encephalitis (199 per m), otitis media (219 per m), upper respiratory infections (222 per m), hepatitis B (349 per m). Compared to other neglected tropical diseases in this combined region, dengue ranks higher than schistosomiasis (4 per m), leprosy (38 per m), trachoma (149 per m), trichuriasis (188 per m), hookworm (191 per m), and ascariasis (209 per m). Dengue ranks just under leishmaniasis (386 per m) and malaria (443 per m) [57].

## Discussion

Our results show that dengue represents a substantial economic and disease burden in SEA. We combined multiple sources of data to quantify this burden. On average, about 52% of the total economic costs of dengue resulted from productivity lost (indirect costs), including non-fatal and fatal cases. The average per capita economic cost of dengue illness represents about 0.03% of the average per capita GDP in the region (in 2010), and total disease burden is 214,000 DALYs per year. Indonesia has a higher share of disease burden than economic burden, which is partly explained by the relatively lower costs per dengue episode.

**Figure 3:**
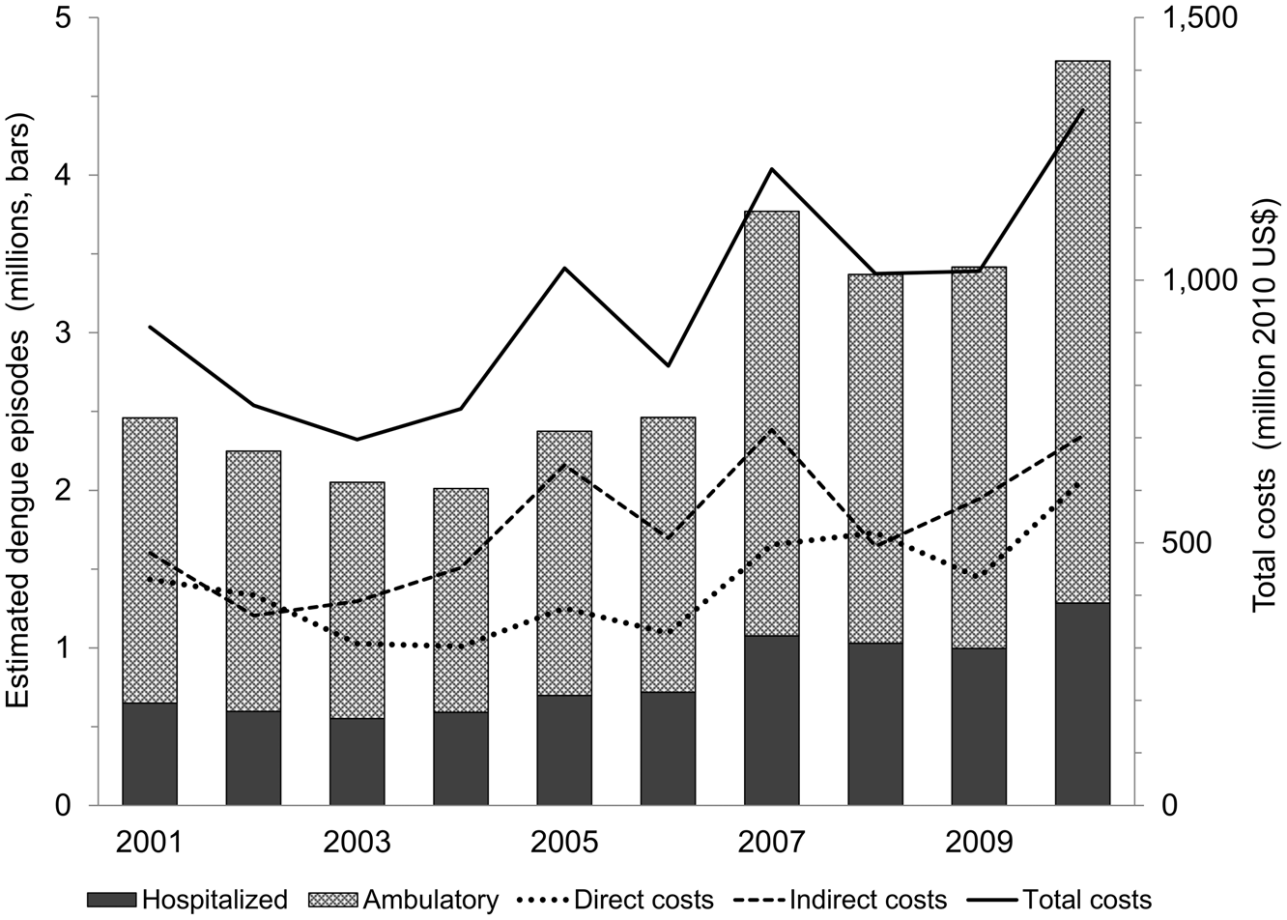
Aggregate values of dengue episodes and economic burden by year for 12 countries in SEA (2001–2010). Source: Authors' calculations.

We used the average number of cases of dengue between 2001 and 2010 to obtain a stable estimate of the burden of dengue, which we consider more useful for policy purposes than an estimate for a specific year. Figure 3 shows the annual variation of total estimated dengue cases and economic burden of dengue in SEA. We are assuming that the EFs and unit costs are constant for all years. As expected, total costs are highly correlated with total number of cases (R^2^ = 0.94, p<0.001); however, the relation depends on which countries are facing an epidemic. While dengue epidemics in the region follow a similar pattern, total costs increase more sharply when the epidemic affects higher-income countries. For example, we estimated fewer dengue episodes in year 2005 (2.37 m) than in 2006 (2.46 m), but because the epidemic affected richer countries in 2005 (e.g., Singapore and Thailand) than in 2006 (e.g., Viet Nam, Indonesia, Cambodia, Philippines), the aggregate costs were higher in 2005 (US$1.02billion) than in 2006 (US$0.84billion). The costs for year 2005 were similar to those in 2008 (US$1.01billion) and 2009 (US$1.02), but the number of cases was much lower in 2005 (2.37 m) than in 2008 (3.37 m) and 2009 (3.42 m), when the dengue epidemic peaked in the poorer countries (e.g., Indonesia, Myanmar).

We found substantial variability in the costs per dengue episode. There was also considerable variability in the country-specific EFs, as has been discussed elsewhere [7]. These variations were addressed using probabilistic analysis; however, costs per episode and EFs remain an area of uncertainty for most of the countries we considered.

Our estimates of economic and disease burden of dengue are consistent with previous estimates from published studies (Table 5). Our estimates of economic burden, without considering costs such as prevention or vector control, for Cambodia, Malaysia, Singapore, and Thailand are higher than in previous studies [2], [16]–[20], and lower than a previous estimate in Viet Nam [21]. Compared to these studies, our higher estimates of economic burden arise mainly because previous studies did not adjust for underreporting of dengue episodes [2], [23], used smaller EFs [16]–[19], considered year intervals with lower reported dengue [18], estimated lower indirect costs [15], estimated productivity loss based on the minimum wage [16], [17], did not consider fatal cases [18], or adjusted for underreporting only of non-fatal cases [20]. Compared to previous estimates of disease burden, our estimates were higher for Myanmar [23], Singapore [20], and Cambodia [15], and lower for Thailand [24], [25]. Our higher estimate for DALYs were partly explained because the previous study for Myanmar only included DHF, did not correct for underreporting, and considered almost 30 years of reporting, which lowered the average reported cases [23], and the estimate for Singapore [20] did not consider an EF for fatal cases of dengue.

**Table 5:** Comparison of estimates of annual economic and disease burden of dengue with previous studies, by country.

Economic burden (US$, million)	Disease burden (DALYs^a^)	Years considered	Source
*Cambodia*			
16.5	15,425	2001–2010	Present study
3.1		2001–2005	Suaya et al., 2009 [2]
8.0	8,243	2006–2008	Beaute and Vong, 2010 [15]
*Malaysia*			
128.0	8,324	2001–2010	Present study
42.4		2001–2005	Suaya et al., 2009 [2]
54.9		2002–2007	Lim et al., 2010 [18]
103.4		2009	Shepard et al. [16], updated 2013 [17]
*Myanmar*			
14.5	13,620	2001–2010	Present study
	3,933^b^	1970–1997	Cho Min Naing, 2000 [23]
*Singapore*			
67.1	1,089	2001–2010	Present study
41.5^c^	734^c^	2000–2009	Carrasco et al.,2011 [20]
*Thailand*			
290.0	28,475	2001–2010	Present study
66.2		2000–2005	Lim et al., 2010 [18]
53.1		2001–2005	Suaya et al., 2009 [2]
126.3		2001–2005	Kongsin et al., 2010 [19]
	31,546	1998–2002	Anderson et al., 2007 [25]
	28,949	2001	Clark et al., 2005 [24]
*Viet Nam*			
23.5	11,079	2001–2010	Present study
30.3		2004–2007	Luong et al., 2012 [21]

a Estimates of the number of disability-adjusted life years (DALYs) were extrapolated to 2010 based on population.

b DALY estimates only include dengue hemorrhagic fever (DHF) episodes.

c The economic and disease burden estimates correspond to Carrasco et al.'s estimates [20], based on the same methods and assumptions than those we used. Economic burden was based on the human capital approach, but Carrasco et al. also estimated annual economic burden of dengue using the friction cost method (US$35.1 million). Similarly, disease burden was estimated using disability weights from previous literature (with an age-weighting constant C = 1), but Carrasco et al. also estimated DALYs using disability weights from WHO and quality of life-based disability weights, and estimated DALYs with C = 1 and C≠1).

The cost per capita associated to dengue in SEA was 68% of that found for the Americas as a whole (US$2.42; range: 1.01–4.47), but DALYs per m were 4.6 times higher than in the Americas (81 DALYs per m; range: 50–131 [3]; WHO's estimate was 73 DALYs per m [57]). This is partly explained by the higher incidence rates of DHF and dengue shock syndrome (DSS) in SEA, which together are approximately 18 times higher than that in the Americas [9], and the case fatality rate is 29 times higher (the estimated case fatality rate was 8/100,000). Also, the main drivers of cost in SEA and the Americas are Indonesia (27% of the total cases of dengue) and Brazil (39% of total cases), respectively. Brazil's GDP per capita is about 3.6 times that of Indonesia's [14] so the average cost per dengue case in the former is substantially higher.

Our estimate of the absolute dengue disease burden of 214,000 DALYs in SEA alone is higher than that of the worldwide disease burden (DALYs) of poliomyelitis (34,000), diphtheria (174,000), or leprosy (194,000) [57]. The DALY rate per population of dengue (372 per million) exceeds that of other diseases of public health importance including Japanese encephalitis, upper respiratory infections, and hepatitis B, and other neglected tropical diseases such as ascariasis, trichuriasis, or hookworm for the combined WHO regions containing SEA.

These results have some limitations and areas of uncertainty. First, the EFs we used to adjust for underreporting were derived from several empirical studies in countries of SEA that used different methodologies (e.g., cohort studies, capture-recapture, hospital records), and some differ in the age groups, or severity of dengue reported [7]. The rate of underreporting also depends on several factors including year of data collection, sample demographics, specific region, vector control activities, disease awareness, quality of the surveillance system. Due to paucity of data, we assumed that the rate of underreporting was constant for each country in SEA during the years considered in this study. Second, we assumed that the average unit costs of inpatient and outpatient treatments of dengue illness were constant across years. Our cost estimates were obtained from empirical studies that in some cases were limited to specific regions or facility types. We could further refine these cost estimates by adjusting other variables such as region, number of specialist physicians, healthcare system, and treatment and technology changes that might have developed since the reference study took place. These levels of detail were not available, but we obtained our estimates from the best accessible data. Third, because there were no studies for all countries in SEA, we had to extrapolate data based on similarities between countries, such as GDP per capita in the case of cost, and an index of healthcare quality for EFs [7].

Fourth, because we lacked more detailed data, we assumed that the age distribution of fatal cases was the same as the age distribution of dengue incidence. This is a conservative assumption, as existing literature suggests that severe episodes of dengue illness in SEA affect mostly infants and children [9], [13], [73], [74], and that children are more vulnerable than adults to shock syndrome [75]. Hence, we would expect the very young to have higher death rates than the rest of the population and therefore, the economic and disease burden might be even higher. Fifth, because the incidence of dengue varies considerably from year to year, we used the average cases of dengue between 2001 and 2010 to obtain more stable estimates. This averaging probably makes our estimates of dengue burden conservative, since several studies indicate that the total number of episodes of symptomatic dengue is increasing [5], [13], [74], [76].

Last, our estimates of the economic and disease burden of dengue illness were based on previous studies that considered the acute symptoms of dengue [2], [77]–[79]. A few recent studies suggest that dengue patients may present long-term symptoms [80]–[84], but there is yet no agreement on the frequency, intensity, or duration of these long-term consequences of dengue infection, sometimes referred to as Dengue Chronic Fatigue Syndrome [83]. If long-term sequelae of dengue are common and affect people's ability to work, then existing studies would be systematically underestimating the economic and disease burden. There was still too much uncertainty over the long-term sequelae of dengue to consider it in our calculations while being conservative. Despite these limitations and areas of uncertainty, we tried to make our estimates of economic and disease burden as accurate as possible considering the limited availability of data.

The most important product of this analysis is estimates of the aggregate and country-specific economic and disease burden of dengue in SEA. These estimates use a consistent methodology that allows comparison among countries and empirically derived adjustments for underreporting. The estimated burden of dengue would have been even higher had we considered other economic costs, such as prevention and vector control [18], [19], [85], [86], disruption of health systems due to seasonal clustering of dengue, decreases in tourism [87], long-term sequelae of dengue [80], [83], or disease complications associated to dengue infection [63], [64], [66], [88]–[92]. Even without counting these additions, our results suggest that exploring new approaches to reduce burden of dengue would be economically valuable.

## Supporting Information

Figure S1 **PRISMA 2009 Flow Diagram.** Source: [48].
(TIF)

Table S1 **PRISMA checklist for literature review.** Note: As this manuscript is not a systematic review nor meta-analysis, the entries in the checklist are limited to those items applicable to this manuscript. Source: [48].
(DOCX)
