# Supplementary figures and images for: Economic and Disease Burden of Dengue in Southeast Asia
Source: PLoS Negl Trop Dis. 2013 Feb 21;7(2):e2055. doi: 10.1371/journal.pntd.0002055 (PMC3578748; doi:10.1371/journal.pntd.0002055)

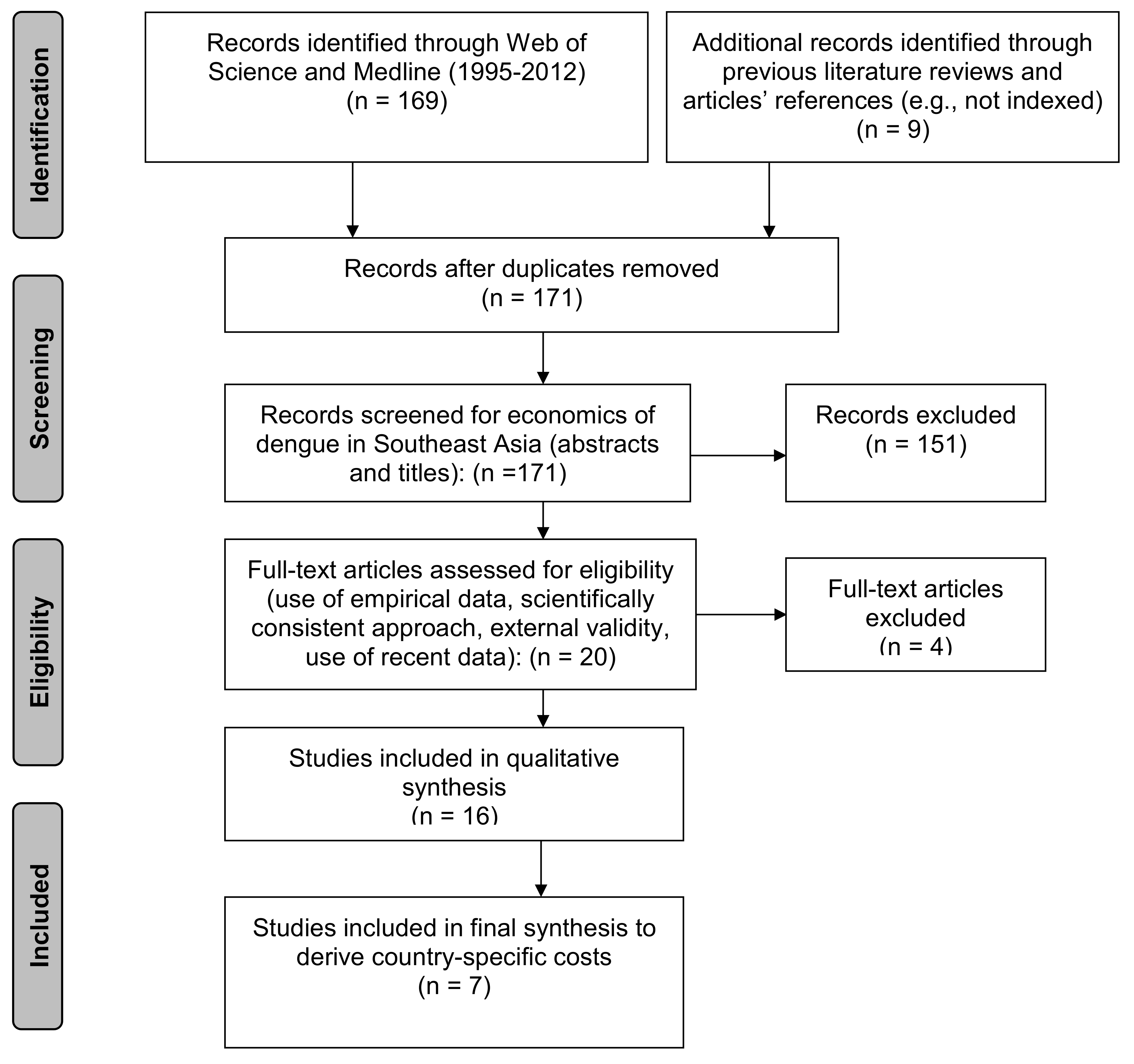

Supplement: Figure S1 — PRISMA 2009 Flow Diagram. Source: [48]. (TIF) [file pntd.0002055.s001.tif]
